# Ipertrofan Revisited—The Proposal of the Complete Stereochemistry of Mepartricin A and B

**DOI:** 10.3390/molecules26185533

**Published:** 2021-09-12

**Authors:** Paweł Szczeblewski, Witold Andrałojć, Justyna Polit, Aneta Żabka, Konrad Winnicki, Tomasz Laskowski

**Affiliations:** 1 Department of Pharmaceutical Technology and Biochemistry and BioTechMed Centre, Faculty of Chemistry, Gdańsk University of Technology, Gabriela Narutowicza Str. 11/12, 80-233 Gdańsk, Poland; pawel.szczeblewski@pg.edu.pl; 2 Institute of Bioorganic Chemistry, Polish Academy of Sciences, Zygmunta Noskowskiego Str. 12/14, 61-704 Poznań, Poland; wandralojc@ibch.poznan.pl; 3 Department of Cytophysiology, Faculty of Biology and Environmental Protection, University of Łódź, Pomorska Str. 141/143, 90-236 Łódź, Poland; justyna.polit@biol.uni.lodz.pl (J.P.); aneta.zabka@biol.uni.lodz.pl (A.Ż.); konrad.winnicki@biol.uni.lodz.pl (K.W.)

**Keywords:** mepartricin, ipertrofan, prostate, NMR, stereochemistry, absolute configuration, aromatic polyene macrolides, partricin, vacidin, gedamycin

## Abstract

Being a methyl ester of partricin, the mepartricin complex is the active substance of a drug called Ipertrofan (Tricandil), which was proven to be useful in treatment of benign prostatic hyperplasia and chronic nonbacterial prostatitis/chronic pelvic pain syndrome. Nevertheless, no direct structural evidence on the stereochemistry of its components has been presented to date. In this contribution, we have conducted detailed, NMR-driven stereochemical studies on mepartricins A and B, aided by molecular dynamics simulations. The absolute configuration of all the stereogenic centers of mepartricin A and B was defined as 3*R*, 7*R*, 9*R*, 11*S*, 13*S*, 15*R*, 17*S*, 18*R*, 19*S*, 21*R*, 36*S*, 37*R*, and 38*S*, and proposed as 41*R*. The geometry of the heptaenic chromophore of both compounds has been established as 22*E*, 24*E*, 26*E*, 28*Z*, 30*Z*, 32*E*, and 34*E*. Our studies on mepartricin ultimately proved that partricins A and B are structurally identical to the previously described main components of the aureofacin complex: gedamycin and vacidin, respectively. The knowledge of the stereochemistry of this drug is a fundamental matter not only in terms of studies on its molecular mode of action, but also for potential derivatization, aiming at improvement of its pharmacological properties.

## 1. Introduction

Mepartricin is a semi-synthetic polyene macrolide complex [1], consisting of two major components, namely mepartricin A and B. Mepartricin is the active substance of a drug called Ipertrofan (Tricandil), which was proven to be useful in treatment of benign prostatic hyperplasia (BPH) [2,3,4] and chronic nonbacterial prostatitis/chronic pelvic pain syndrome (CPPS) [5]. Mepartricin has been demonstrated to increase the fecal excretion of estrogen in rats [6]. It also improved the International Prostatic Symptom Score of BPH patients while reducing the serum concentration of estrogen [7]. Moreover, this drug was found to cause softening of the prostate tissue in dogs with spontaneous BPH, also stimulating substantial improvements in hyperplastic tissue. On the basis of the aforementioned experimental findings, it has been assumed that mepartricin reduces the effects of estrogen on the prostate gland and therefore expresses anti-BPH activity [8]. Similarly to all members of the heptaenic family of polyene macrolides, mepartricin effectively binds to steroids [9]. This feature is being considered as the most probable molecular foundation of the therapeutic effect.

Mepartricin is in fact a methyl ester of partricin [10], an antibiotic complex produced by *Streptomyces aureofaciens NRRL 3878*, which belongs to a subgroup of aromatic heptaene macrolides. Members of that family generally exhibit substantially higher antifungal activity than the golden standard in the treatment of systemic fungal infections, the non-aromatic heptaene macrolide amphotericin B (AmB) [11].

Although mepartricin is still a registered drug in several countries, only the gross structure of mepartricins A and B were reported [12] and claimed to be identical with the gross structure of the methyl esters of the antibiotics gedamycin and vacidin, respectively [13]. No direct stereochemical studies on any of the partricins and/or their derivatives could be traced in the literature. Moreover, mepartricins are sometimes misrepresented as *all-trans* compounds—i.e., containing heptaenic chromophore of seven *E* double bonds. Since it is obvious that rational studies on the molecular mode of action of mepartricins require full knowledge on their stereochemistry, we strongly believe that this matter deserves full clarification.

**Figure 1:**
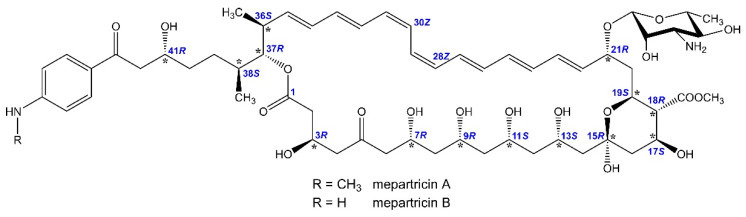
Stereochemistry of mepartricin A and B.

Therefore, herein we report NMR-evidenced and molecular-modelling-aided stereochemistry assignment of Ipertrofan active agents: mepartricins A and B—i.e., the methyl esters of partricin A and B, respectively (Figure 1). In addition, since various pieces of data were reported for both partricins and their derivatives under different names [12,13,14,15,16], we also present a synthesis of structural data and propose a unification of nomenclature for these compounds.

## 2. Results

### 2.1. A Brief Introduction to the Procedure

In order to establish the complete stereochemistry of mepartricin A and B, a set of 2D NMR experiments—DQF-COSY, TOCSY, ROESY, HSQC, and HMBC—was conducted for both compounds (please consult Figures S1–S14). The use of ROESY instead of a NOESY experiment was dictated by the negligible intensity of the NOE cross-peaks for the molecules under study, due to unfavorable molecular weight regime. This statement is actually true for most heptaene macrolides—in terms of two-dimensional dipolar couplings’ studies, it is ROESY or nothing. Yet, it must be noted that in general ROESY has inherently lower sensitivity than a NOESY experiment and one needs to be cautious during its interpretation, due to a possibility of registration of TOCSY-like cross peaks.

This procedure was previously successfully applied for the definition of stereochemistry of almost 20 polyene macrolides and their derivatives, including: the candicidins [17,18,19,20], rimocidin [21], candidin [22], mycoheptin A2 [23], etc. In this standardized approach, DQF-COSY and TOCSY experiments have allowed tracing of connectivities within isolated proton spin systems, separated by quaternary carbon atoms and oxygen-involving lactone and glycosidic bonds. 2D-^1^H,^13^C-HMBC experiment has enabled merging those structural blocks into a gross structure of the antibiotic via long-range heteronuclear couplings. Finally, absolute configuration of the stereogenic centers of a studied compound has been revealed by an appropriate set of vicinal proton–proton coupling constants, accompanied by relevant dipolar couplings revealed by the ROESY experiment.

It should be stated that the stereochemistry of all but one (see below) chiral carbons of the macrolide is actually being established in relation to the absolute configuration of the hydroxymethine carbon atom C21, glycosidically binding a monosaccharide moiety of previously defined absolute stereochemistry. If a monosaccharide bound to a macrolactone ring—usually a d-aminosugar—produces dipolar couplings to the protons of the aglycone, these ROEs unambiguously define the absolute configuration of the aforementioned hydroxymethine carbon. This observation, which was first reported during the model studies on amphotericin B [24], has laid a foundation for a general method of the elucidation of the absolute configuration of chiral secondary alcohols [25,26].

### 2.2. Assignment of the Stereochemistry of Mepartricin A and B

**Figure 2:**
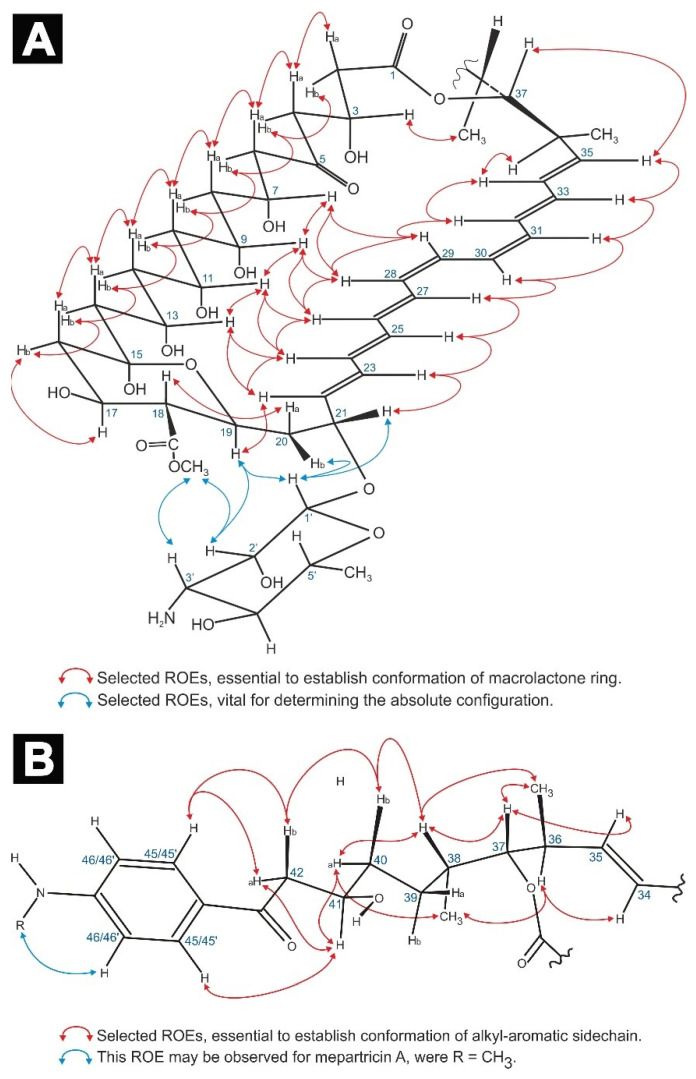
Selected ROEs, aiding the definition of the average conformation of the macrolactone ring (**A**) and alkyl-aromatic sidechain (**B**), as well as the relative and absolute configuration of all stereogenic centers of the studied molecules.

Previous structural studies on mepartricin A and B have proven that—in case of both molecules—a d-mycosamine moiety was glycosidically bound to the C21 atom of the aglycone [12,13]. Our experiments additionally revealed the presence of H1′/H19, H1′/H20b, H1′/H21, H2′/COOMe, and H3′/COOMe dipolar couplings in the ROESY spectra of both compounds (Figure 2A). These ROEs unambiguously defined the stereochemistry of C21 as 21*R*. This assignment, along with the measured vicinal coupling constants: ^3^*J*_H20b/H21_ = 5.6 Hz, ^3^*J*_H19/H20a_ = 10.5 Hz, ^3^*J*_H18/H19_ = 10.2 Hz, ^3^*J*_H17/H18_ = 10.1 Hz (last three ^3^*J* values characteristic for diaxial coupling) and observed ROE pathways: H18/H20a, H19/H22, and H20b/H21/H23 enabled a straightforward definition of the absolute configuration of C17, C18, and C19 as 17*S*, 18*R*, and 19*S*.

Following that road, a set of two long ROE pathways was observed: H2b/H4b/H6b/H8b/H10b/H12b/H14b/H16b and H2a/H4a/H6a/H8a/H10a/H12a/H14a/H16a, accompanied by ROEs H7/H9/H11/H13, H22/H13/H24, H24/H11/H26, etc. (see Figure 2A). Moreover, DQF-COSY spectrum revealed a consistent pattern regarding the scalar couplings within the C6-C14 fragment: large vicinal coupling constant between a methine proton Hn and a methylene proton H(n ± 1)b, followed by a small vicinal coupling constant between a methine proton Hn and a methylene proton H(n ± 1)a (see the Appendix A section: Table A1). For instance, proton H13 exhibited large ^3^*J*s to H12b and H14b, while displaying small ^3^*J*s to protons H12a and H14a, etc. All these data combined, in relation to 17*S*, established the stereochemistry of C7, C9, C11, C13, and C15 as 7*R*, 9*R*, 11*S*, 13*S*, and 15*R*.

Before assigning the absolute configuration of the remaining asymmetric centers (C3, C36, C37, C38, and C41), the *E*-*Z* geometries of the carbon atoms within the chromophore region (C22-C35) had to be established. All the measured coupling constants within the double bonds, except C28-C29 and C30-C31, were in a range within 15.2–15.5 Hz, indicating the *E* geometries. The ^3^*J*_H28/H29_ and ^3^*J*_H30/H31_ coupling constants were equal to 11.5 Hz and 11.3 Hz, respectively, which pointed to *Z* geometries of those double bonds. These assignments were strongly supported by uninterrupted ROE pathways: H22/H24/H26/H28/H29/H32/H34 and H23/H25/H27/H30/H31/H33/H35 (Figure 2A), as well as ~5 ppm shielding of C27, C29, C30, and C32 carbons in ^13^C-NMR spectrum, resulting from C27/C30 and C29/C32 ɣ-effects. Hence, the geometry of the heptaenic chromophore of both mepartricins was established as 22*E*, 24*E*, 26*E*, 28*Z*, 30*Z*, 32*E*, and 34*E*.

Given the geometry of the chromophore and all the previously established absolute configurations, the acquired spectral data, i.e., vicinal coupling constants ^3^*J*_H36/H37_ = 9.9 Hz and ^3^*J*_H37/H38_ = 2.3 Hz—as well as ROEs H3/H34, H3/Me38, H34/H36, H35/H37, H36/Me38, and H38/Me36—pointed out the relative configuration of C3, C36, C37, and C38; as 3R*, 36S*, 37R*, and 38S*. The absolute configurations in the C36-C3 region were deduced based upon the fact that only one enantiomer of this fragment could close the macrolactone ring without creating severe distortions within the C6-C14 region, which would break the previously mentioned H2a to H14a and H2b to H14b ROE pathways (Figure 2A). Hence, it was possible to assign the absolute configurations of the aforementioned chiral centers as 3*R*, 36*S*, 37*R*, and 38*S*.

The absolute configuration of C41 could not be related to the stereochemistry of C36-C38 region in a straightforward manner due to perfect, yet unfortunate superposition of H39a and H39b resonances. Therefore, vicinal coupling constants within the C38-C39 and C39-C40 bonds could not be measured. Hence, protons H40a/b and H42a/b could not be unambiguously assigned in the ^1^H-NMR spectrum without the knowledge on C41′s absolute configuration. To solve that issue, we have performed extensive molecular dynamics simulations of atomistic models of 41*R* and 41*S*, possible epimers of mepartricin A. The resulting trajectories have revealed the formation of 41OH/43CO hydrogen bond in case of both epimers (Figure S15 and S16), which significantly decreased the conformational freedom within the C41-C43 region. On the basis of the simulations performed, we have calculated theoretical values of vicinal coupling constants in H40a/H41, H40b/H41, H41/H42a, and H41/H42b pairs, using time-averaged Haasnoot–de Leeuw–Altona (HLA) equation [27]. The results, presented in Table 1, were in very good agreement with the 41*R* epimer (average disagreement between calculated and experimental ^3^*J* values of > 0.3 Hz; maximum disagreement of 0.7 Hz), while in case of 41*S* alternative the differences between theoretical and measured values were much more pronounced (average disagreement of 2.0 Hz; maximum disagreement of 3.3 Hz). Therefore, the absolute configuration at C41 was proposed to be 41*R*.

The above proposal of C41′s absolute configuration was strongly supported by the fact that (so far) all stereostructurally elucidated aromatic heptaene macrolides, containing alkyl-aromatic sidechain with hydroxylic moiety attached to C41, exhibited identical stereochemistry of this asymmetric center [17,20]. Moreover, recent studies have shown that this regularity results from the shared biosynthetic pathway among the species producing heptaenic macrolides [28]. Yet, in case of the candicidins, this spectroscopic assignment was much more straightforward due to the presence of a methyl moiety at C40 [18], which both mepartricins lack. Hence, it should be stated that the herein presented investigation of the absolute configuration of C41, although most likely correct, has not resulted in an unambiguous proof and, therefore, could require more robust confirmation by chemical and spectroscopic methods in the future.

As a result of the above insight, protons H40a, H40b, H42a, and H42b were assigned, which allowed the definition of the averaged conformation of the alkyl-aromatic sidechain of mepartricin A and B, presented in Figure 2B.

## 3. Discussion

Although mepartricin is an active substance of a drug called Ipertrofan (Tricandil), no structural evidence on the stereochemistry of its components has been reported to date. In this contribution, we have conducted detailed, NMR-driven stereochemical studies on mepartricins A and B, aided by molecular dynamics simulations. The absolute configuration of all the stereogenic centers of mepartricin A and B was defined as 3*R*, 7*R*, 9*R*, 11*S*, 13*S*, 15*R*, 17*S*, 18*R*, 19*S*, 21*R*, 36*S*, 37*R*, and 38*S*, and proposed to be 41*R*. The geometry of the heptaenic chromophore of both compounds has been established as 22*E*, 24*E*, 26*E*, 28*Z*, 30*Z*, 32*E*, and 34*E*. Since, as stated before, mepartricins A and B are the methyl esters of partricin A and B, respectively, it turned out that not only the gross structure [13]—but also the stereochemistry of partricin A—is identical to the antibiotic gedamycin [16], while the gross structure and stereochemistry of partricin B is identical to vacidin [14,15]. Therefore, we state that the names ‘partricin A’ and ‘gedamycin’ refer to the same molecule and henceforth should be used as synonyms, while the same case occurs for the names ‘partricin B’ and ‘vacidin’.

It is obvious that the knowledge on the stereochemistry of a drug is a fundamental matter not only in terms of studies on its molecular mode of action, but also for potential derivatization, aiming at improvement of its pharmacological properties. Moreover, it should be noted that, recently, we have reported that candicidin D—the most widely known aromatic polyene macrolide antifungal antibiotic—undergoes photochemical isomerization while exposed to direct sunlight, resulting in straightening of its heptaenic chromophore [29]. Similar behavior was also previously suggested for the partricins [30], which is currently under our detailed investigation. This phenomenon—when proved—should be considered as an additional variable and possibly taken into account during the formulation process of a drug.

## 4. Materials and Methods

### 4.1. NMR Spectroscopy

The NMR spectra were recorded on a 700 MHz Bruker Avance III HD spectrometer (Bruker GmbH, Karlsruhe, Germany) equipped with a QCI CryoProbe. All the experiments were performed in solvent system pyridine-*d_5_*-methanol-*d_4_*, 9:1 (*v*/*v*) at ambient temperature with a sample concentration of 10 mg/ml. Chemical shifts are reported in *δ*_H_ (ppm) units using ^1^H residual resonance of pyridine-*d_5_* (7.19 ppm) as internal standard. The 1D ^1^H-NMR spectra were collected with a digital resolution of 0.5 Hz. The ^1^H 90º pulse length was 7.6 µs.

2D-^1^H,^1^H spectra were measured in the phase-sensitive mode with a spectral width of 7705 Hz.

DQF-COSY spectra were acquired in a 4096 × 512 matrix with 32 accumulations per increment and was processed in a 4K × 2K matrix.

TOCSY spectra were acquired with a mix time of 60 ms in a 2048 × 512 matrix with 32 accumulations per increment and was processed in a 2K × 1K matrix.

ROESY spectra were acquired with a mix time of 350 ms in a 2048 × 512 matrix with 72 accumulations per increment and was processed in a 2K × 1K matrix.

2D-^1^H,^13^C-HSQC and 2D-^1^H,^13^C-HMBC experiments were performed with pulse field gradients.

HSQC and edited-HSQC spectra were acquired in the phase-sensitive mode with ^1^*J*_(CH)_ set to 140 Hz. The spectral windows for ^1^H and ^13^C axes were 7705 Hz and 29,177 Hz, respectively. Data were collected in a 2048 × 256 matrix with 64 accumulations per increment and processed in a 2K × 1K matrix.

HMBC spectra were acquired in absolute value mode with ^n^*J*_(CH)_ set to 9 Hz. The spectral windows for ^1^H and ^13^C axes were 7705 Hz and 40515 Hz, respectively. The data were collected in a 2048 × 256 matrix with 184 accumulations per increment and processed in a 2K × 1K matrix.

### 4.2. Molecular Modeling Studies

Molecular dynamics. Parameters for the molecular models of 41*R*-mepartricin A (**41R-MA**) and 41*S*-mepartricin A (**41S-MA**) were taken from CHARMM36 Generalized Force Field [31]. Partial atomic charges and dihedral definitions were refined using GAUSSIAN09 software (Gaussian Inc., version 09, revision D.01, Wallingford CT, USA) [32] (MP2/6-31G* level of theory) and ForceField Tool Kit (ffTK) as a part of VMD 1.9.4 [33]. **41R-MA** and **41S-MA** were then solvated in pyridine cubic boxes (1540 and 1542 pyridine molecules, respectively). Pyridine was chosen as a simulation environment, due to the fact that it was used as an NMR solvent; its parameters were taken from CHARMM36 Generalized Force Field [31]. After initial equilibration, both systems were subjects to 200-ns long MD runs. All the simulations were carried out using GROMACS software (The GROMACS development teams at the Royal Institute of Technology and Uppsala University, version 2020.4, Stockholm/Uppsala, Sweden) [34] using leapfrog scheme with a time step of 2 fs. The particle mesh Ewald technique with a cutoff of 1 nm and a grid spacing of approx. 0.1 nm was employed to evaluate the electrostatic forces. The van der Waals interactions were calculated using a Lennard-Jones potential with a cutoff of 1 nm. The simulation was conducted at a constant temperature of 300 K and a constant pressure of 1 bar using the weak coupling method with relaxation times of 0.1 ps and 0.5 ps, respectively. All of the covalent bonds’ lengths were constrained using the P-LINCS and SETTLE algorithms.

### 4.3. Haasnoot–de Leeuw–Altona (HLA) Equation

After completion of the both MD simulations (**41R-MA** and **41S-MA**), the representative statistical probes of 2000 frames for each variant were extracted from the resulting trajectories. For each frame, the ^3^*J*_H40a/H41_, ^3^*J*_H40b/H41_, ^3^*J*_H41/H42a_, and ^3^*J*_H41/H42b_ vicinal coupling constants were calculated using HLA equation [27] (Equation 1)

^3^*J* = P_1_cos^2^ϕ + P_2_cosϕ + P_3_ + ∑∆χ_i_[P_4_ + P_5_cos^2^(ξ_i_ϕ + P_6_|∆χ_i_|)] (1)

The resulting 2000 ^3^*J* coupling values for each dihedral angle of interest were then averaged for all snapshots from the 500 ns MD simulations, yielding the presented ^3^*Ĵ*_H40a/H41_, ^3^*Ĵ*_H40b/H41_, ^3^*Ĵ*_H41/H42a_, and ^3^*Ĵ*_H41/H42_ ensemble averages.

The parameters P1, P2, P3, P4, P5, and P6 of the used HLA equation were equal to: 14.64, −0.78, 0.58, 0.34, −2.31, and 18.40, respectively, and were taken from the MestReJ software [35].

The ξ_i_ parameters were equal to 1 or −1, depending on the orientation of each S_i_ substituent [27].

The Δχ_i_ parameters were extracted from the MestReJ software [35]. They depended on the nature of each Si substituent and were equal to: 0 for H, 0.62 for CHMeOH, 0.72 for CH_2_C(O)R, 0.76 for CH_2_CH_2_R, and 1.33 for OH.

## Figures and Tables

**Table 1:** Measured H40a/H41, H40b/H41, H41/H42a, and H41/H42b vicinal coupling constants compared to the theoretical values, calculated for 41*R* and 41*S* diastereoisomers of mepartricin A.

Coupling Protons (i,j)	Measured ^3^*J*_i/j_ (Hz)	Calculated ^3^*Ĵ*_i/j_ (Hz)		Absolute Difference between the Experimental and Calculated ^3^*J*_i/j_ (Hz)	
		41*R*	41*S*	41*R*	41*S*
H40a, H41	4.5	3.8	2.3	0.7	2.2
H40b, H41	8.5	8.7	9.7	0.2	1.2
H41, H42a	3.2	3.1	4.5	0.1	1.3
H41, H42b	9.3	9.2	6.0	0.1	3.3

## Data Availability

The data presented in this study, including high resolution NMR spectra and MD trajectories, are available on request from the corresponding author. The data are not publicly available due to the large size of the respective files.

## Supplementary Materials

The following are available online. Figure S1: ^1^H-NMR spectrum of mepartricin A; Figure S2: ^1^H-NMR spectrum of mepartricin B; Figure S3: ^13^C NMR spectrum of mepartricin A; Figure S4: ^13^C-NMR spectrum of mepartricin B; Figure S5: 2D-^1^H,^1^H-DQF-COSY spectrum of mepartricin A; Figure S6: 2D-^1^H,^1^H-DQF-COSY spectrum of mepartricin B; Figure S7: 2D-^1^H,^1^H-TOCSY spectrum of mepartricin A; Figure S8: 2D-^1^H,^1^H-TOCSY spectrum of mepartricin B; Figure S9: 2D-^1^H,^1^H-ROESY spectrum of mepartricin A; Figure S10: 2D-^1^H,^1^H-ROESY spectrum of mepartricin B; Figure S11: Edited 2D-^1^H,^13^C-HSQC spectrum of mepartricin A; Figure S12: 2D-^1^H,^13^C-HMBC spectrum of mepartricin A; Figure S13: Edited 2D-^1^H,^13^C-HSQC spectrum of mepartricin B; Figure S14: 2D-^1^H,^13^C-HMBC spectrum of mepartricin B; Figure S15: Average structures of the alkyl-aromatic sidechains of the possible 41*R*-epimers of mepartricins A and B; Figure S16: Average structures of the alkyl-aromatic sidechains of the possible 41*S*-epimers of mepartricins A and B.

## Sample Availability

Samples of the compounds are not available from the authors.

## Appendix A

**Table A1:** ^1^H and ^13^C-NMR spectroscopic data for mepartricin A and B (700 MHz, C_6_D_5_N/CD_3_OD (9:1, *v*/*v*)). In every column, letter A stands for spectroscopic data for mepartricin A, letter B stands for the analogous value for mepartricin B, whereas no letter given means that the presented data is identical for both antibiotics.

Mepartricin A and B					
Position	*δ*_C_, Type		*δ*_H_	*J*_H,H_ (Hz)	ROE Contacts
*Aglycone*					
1	170.88,	C	–	–	–
2a	43.67,	CH_2_	2.664	15.4 (2b), 9.2 (3)	2b, 3, 4a
2b			2.948	15.4 (2a), 3.4 (3)	2a, 3, 4b
3	63.82,	CH	4.963	9.2 (2a), 3.4 (2b), 8.5 (4a), 5.0 (4b)	2a, 2b, 4a, 4b, 34, Me38
4a	51.34,	CH_2_	2.836	8.5 (3), 17.2 (4b)	2a, 3, 4b
4b			3.099	5.0 (3), 17.2 (4b)	2b, 3, 4a
5	208.49,	C	–	–	–
6a	51.30,	CH_2_	2.558	16.5 (6b), 1.9 (7)	4a, 6b, 7
6b			2.821	16.5 (6a), 9.6 (7)	4b, 6a, 7
7	67.40,	CH	4.698	1.9 (6a), 9.6 (6b), 2.0 (8a), 9.4 (8b)	6a, 6b, 8a, 8b, 9, 28, 29
8a	43.86,	CH_2_	1.334	2.0 (7), 13.5 (8b), 1.9 (9)	6a, 7, 8b, 9, 10a
8b			1.773	9.4 (7), 13.5 (8b), 9.8 (9)	6b, 7, 8a, 10b
9	73.04,	CH	4.217	1.9 (8a), 9.8 (8b), 2.0 (10a), 10.0 (10b)	7, 8a, 10a, 11, 26, 28
10a	44.34,	CH_2_	1.406	2.0 (9), 14.0 (10b), 1.8 (11)	8a, 9, 10b, 11, 12a
10b			1.668	10.0 (9), 14.0 (10a), 10.3 (11)	8b, 10a
11	73.82,	CH	4.269	1.8 (10a), 10.3 (10b), 2.1 (12a), 9.4 (12b)	9, 10a, 12a, 13, 24, 26
12a	44.09,	CH_2_	1.357	2.1 (11), 13.3 (12b), 1.9 (13)	10a, 11, 12b, 13, 14a
12b			1.677	9.4 (11), 13.3 (12a), 9.9 (13)	12a, 14b
13	69.36,	CH	4.751	1.9 (12a), 9.9 (12b), 2.1 (14a), 9.5 (14b)	11, 12a, 14a, 22, 24
14a	46.68,	CH_2_	1.730	2.1 (13), 14.5 (14b)	12a, 13, 14b
14b			1.945	9.5 (13), 14.5 (14a)	12b, 14a, 16b
15	98.01,	C	–	–	–
16a	45.28,	CH_2_	1.734	12.4 (16b), 10.4 (17)	16b, 18
16b			2.536	12.4 (16a), 4.3 (17)	14b, 16a, 17
17	66.34,	CH	5.006	10.4 (16a), 4.3 (16b), 10.1 (18)	16b, 18, 19
18	58.59,	CH	2.809	10.1 (17), 10.2 (19)	16a, 17, 19, 20a
19	66.79,	CH	5.082	10.2 (18), 10.5 (20a)	17, 18, 20b, 22, 1′, 2′
20a	37.72,	CH_2_	1.999	10.5 (19), 15.8 (20b)	18, 20b, 21
20b			2.447	15.8 (20a), 5.6 (21)	19, 20a, 21, 1′
21	75.70,	CH	4.936	5.6 (20b), 9.1 (22)	20a, 20b, 22, 23, 1′
22	137.00,	CH	6.507	9.1 (21), 15.4 (23)	13, 19, 21, 24
23	129.99,	CH	6.392	15.4 (22), 10.9 (24)	21, 25
24	134.31,	CH	6.753	10.9 (23), 15.5 (25)	11, 13, 22, 26
25	132.85,	CH	6.544	15.5 (24). 11.2 (26)	23, 27
26	135.21,	CH	6.789	11.2 (25), 15.2 (27)	9, 11, 24, 28
27	125.11,	CH	7.048	15.2 (26), 11.0 (28)	25, 30
28	130.49,	CH	6.616	11.0 (27), 11.5 (29)	7, 9, 26, 29
29	128.25,	CH	7.052	11.5 (28), 10.8 (30)	7, 28, 32
30	124.88,	CH	6.736	10.8 (29). 11.3 (31)	27, 31
31	130.47,	CH	6.275	11.3 (30), 10.9 (32)	30, 33
32	128.11,	CH	7.261	10.9 (31), 15.5 (33)	29, 34
33	134.10,	CH	6.370	15.5 (32), 11.2 (34)	31, 35
34	133.17,	CH	6.489	11.2 (33), 15.5 (35)	3, 32, 36
35	137.63,	CH	5.610	15.5 (34), 9.3 (36)	33, 36, 37, Me36
36	40.05,	CH	2.592	9.3 (35), 9.9 (37)	34, 35, 37, 39ab, Me36, Me38
37	78.29,	CH	5.109	9.9 (36), 2.3 (38)	35, 36, 38, 39ab, Me36, Me38
38	33.88,	CH	1.948	2.3 (37), ? (39ab)*	37, 39ab, 40a, 40b, 41, Me36, Me38
39ab*	30.77,	CH_2_	A: 1.801 B: 1.799	? (38, 40a, 40b)*	36, 37, 38, 40a, 40b, 41, Me36, Me38
40a	A: 35.65, B: 35.67,	CH	A: 1.918 B: 1.902	? (39ab)*, 15.9 (40b), 4.5 (41)	38, 39ab, 40b, 41, 42a, 42b, Me38
40b			A: 1.853 B: 1.854	? (39ab)*, 15.9 (40a), 8.5 (41)	38, 39ab, 40a, 42a, 42b
41	A: 68.35, B: 68.30,	CH	A: 4.589 B: 4.572	4.5 (40a), 8.5 (40b), 3.2 (42a), 9.3 (42b)	38, 39ab, 40a, 42a, 45/45′
42a	A: 46.00, B: 45.95,	CH_2_	A: 3.204 B: 3.183	3.2 (41), 15.4 (42b)	40a, 40b, 41, 42b, 45/45′
42b			A: 3.390 B: 3.366	9.3 (41), 15.4 (42a)	40a, 40b, 42a, 45/45′
43	A: 197.72, B: 197.64,	C	–	–	–
Me36	16.26,	CH_3_	0.971	6.7 (36)	35, 36, 37, 38, 39ab
Me38	13.01,	CH_3_	1.053	6.5 (38)	3, 36, 37, 38, 39ab, 40a
COOMe	174.05,	C	–	–	–
COOMe	51.38,	CH_3_	3.696	–	2′, 3′
NHMe	A: 29.27, B: –,	CH_3_	A: 2.791 B: –	–	46/46′
*Aromatic Moiety*					
45/45′	A: 131.04, B: 131.21,	CH	8.148	8.7 (46/46′)	41, 42a, 42b, 46/46′
46/46′	A: 110.91, B: 113.23,	CH	6.783	8.7 (45/45′)	45/45′, NHMe
C*CO	A: 154.39, B: 154.21,	C	–	–	–
C*NH	A: 126.16, B: 126.69,	C	–	–	–
*Mycosamine Moiety*					
1’	98.34,	CH	4.906	1.8 (2′)	2′, 3′, 5′, 19, 20b, 21
2’	71.19,	CH	4.439	1.8 (1′), 3.4 (3′)	1′, 3′, 19, COOMe
3’	57.91,	CH	3.120	3.4 (2′), 9.6 (4′)	1′, 2′, 5′, COOMe
4’	74.19,	CH	3.806	9.6 (3′), 9.8 (5′)	6′
5’	74.36,	CH	3.669	9.8 (4)’, 6.1 (6′)	1′, 3′, 6′
6’	18.37,	CH_3_	1.589	6.1 (5′)	4′, 5′

* These protons were perfectly superimposed, hence the values of the coupling constants involving protons 39a and 39b could not be measured.
