# Supplementary material for: Ipertrofan Revisited—The Proposal of the Complete Stereochemistry of Mepartricin A and B
Source: Molecules. 2021 Sep 12;26(18):5533. doi: 10.3390/molecules26185533 (PMC8467382; doi:10.3390/molecules26185533)
Supplement: Supplementary file 1 [file molecules-26-05533-s001.zip › molecules-1364799-supplementary.pdf]

Ipertrofan Revisited—The Proposal of the Complete Stereochemistry of Mepartricin A and B

Paweł Szczeblewski[1], Witold Andrałojć[2], Justyna Polit[3], Aneta Żabka[3], Konrad Winnicki[3] and Tomasz Laskowski[1,*]

[1] Department of Pharmaceutical Technology and Biochemistry and BioTechMed Centre, Faculty of Chemistry, Gdańsk University of Technology, Gabriela Narutowicza Str. 11/12, 80-233 Gdańsk, Poland; pawel.szczeblewski@pg.edu.pl

[2] Institute of Bioorganic Chemistry, Polish Academy of Sciences, Zygmunta Noskowskiego Str. 12/14, 61-704 Poznań, Poland; wandralojc@ibch.poznan.pl

[3] Department of Cytophysiology, Faculty of Biology and Environmental Protection, University of Łódź, Łódź, Pomorska Str. 141/143, 90-236, Poland; justyna.polit@biol.uni.lodz.pl (J.P.); aneta.zabka@biol.uni.lodz.pl (A. Ż.); konrad.winnicki@biol.uni.lodz.pl (K.W.)

* Correspondence: tomasz.laskowski@pg.edu.pl; Tel.: +48 58 347 20 79

SUPPLEMENTARY INFORMATION

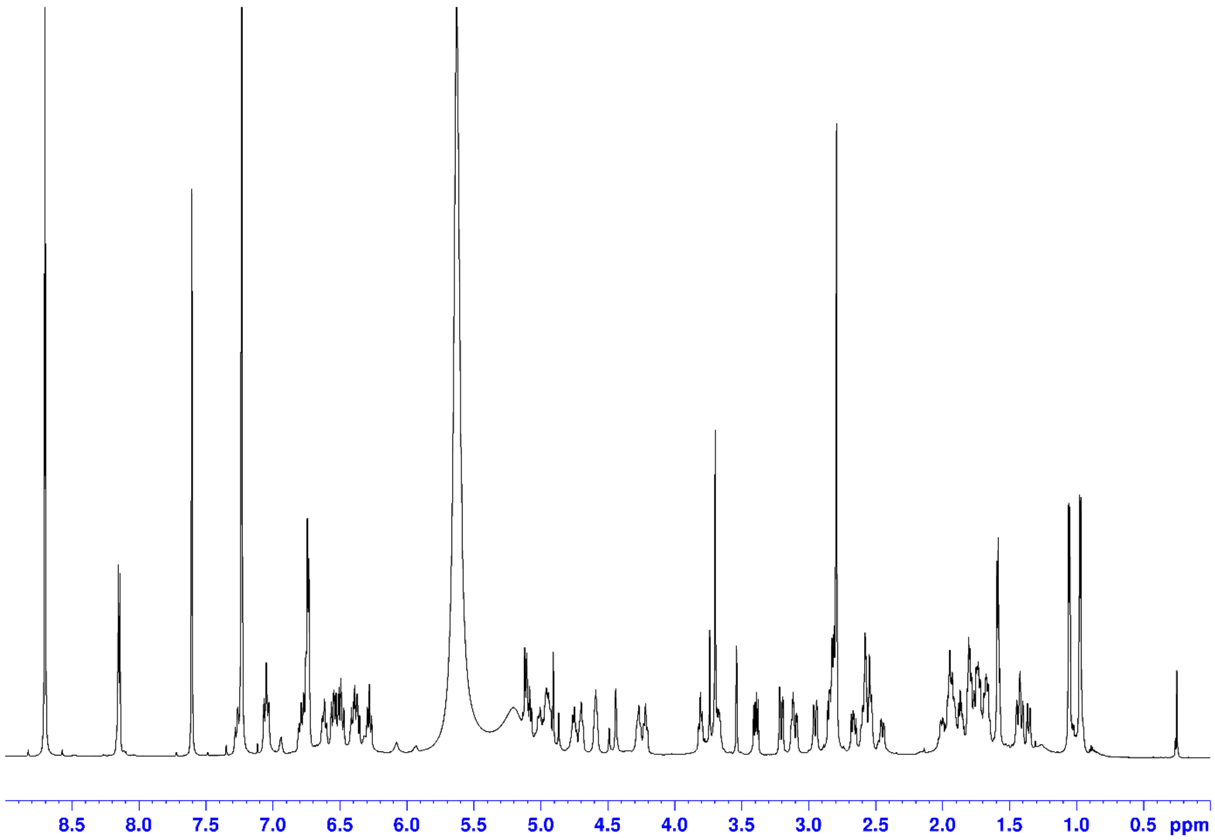

Figure S1. ^{1}H NMR spectrum of mepartricin A.

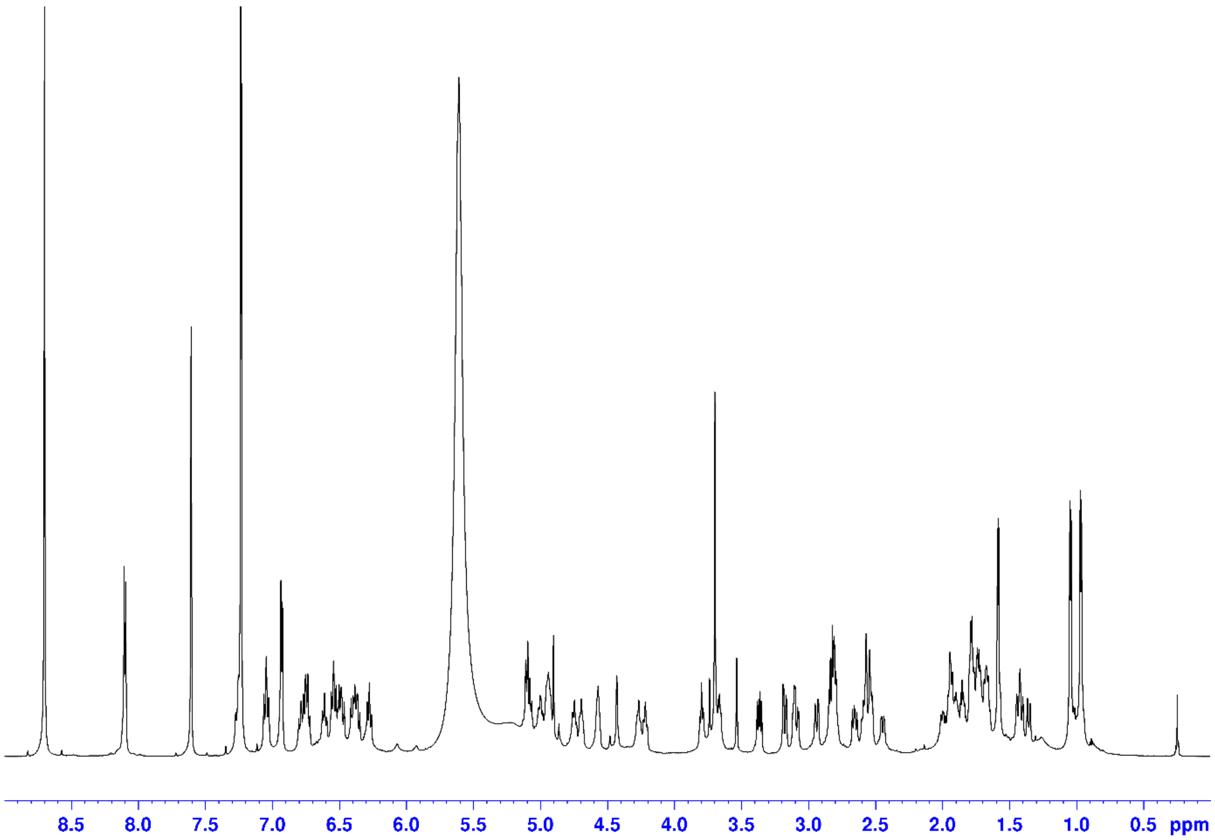

Figure S2. ^{1}H NMR spectrum of mepartricin B.

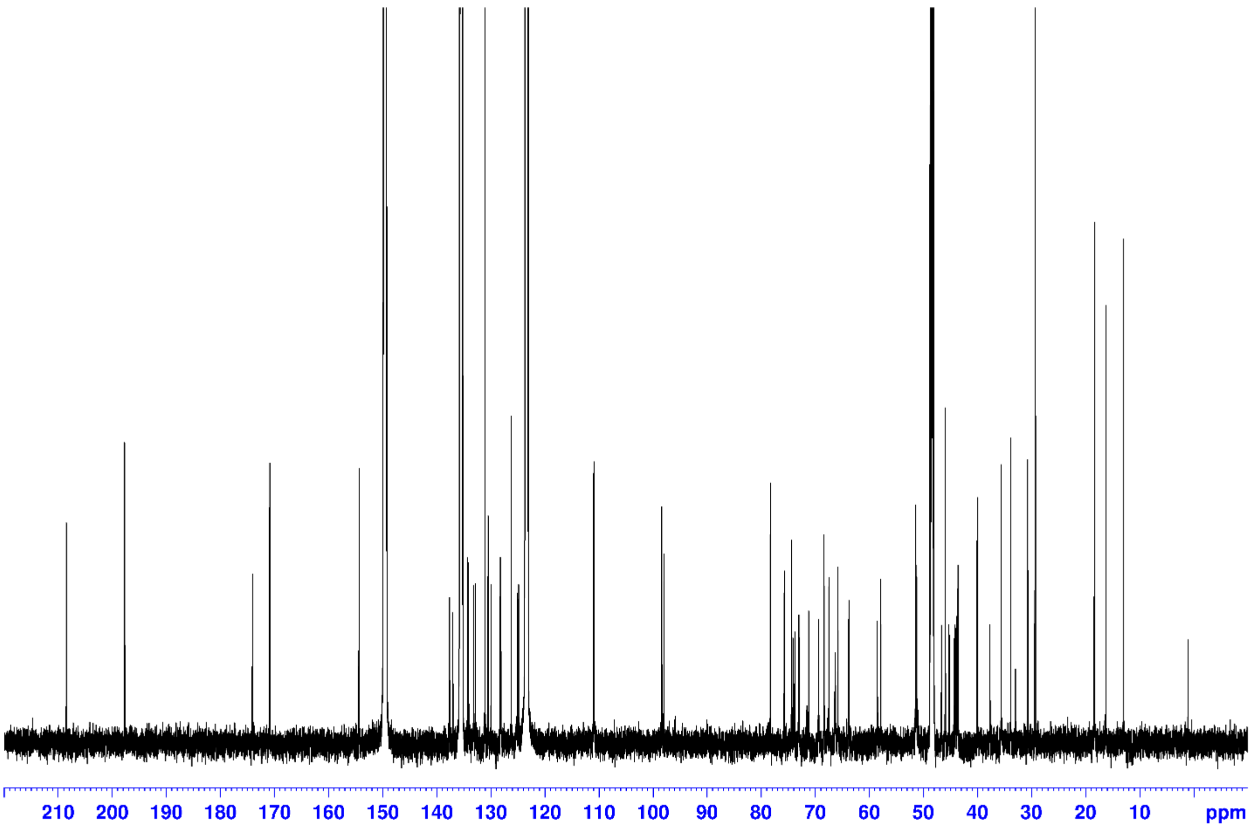

Figure S3. ^{13}C NMR spectrum of mepartricin A.

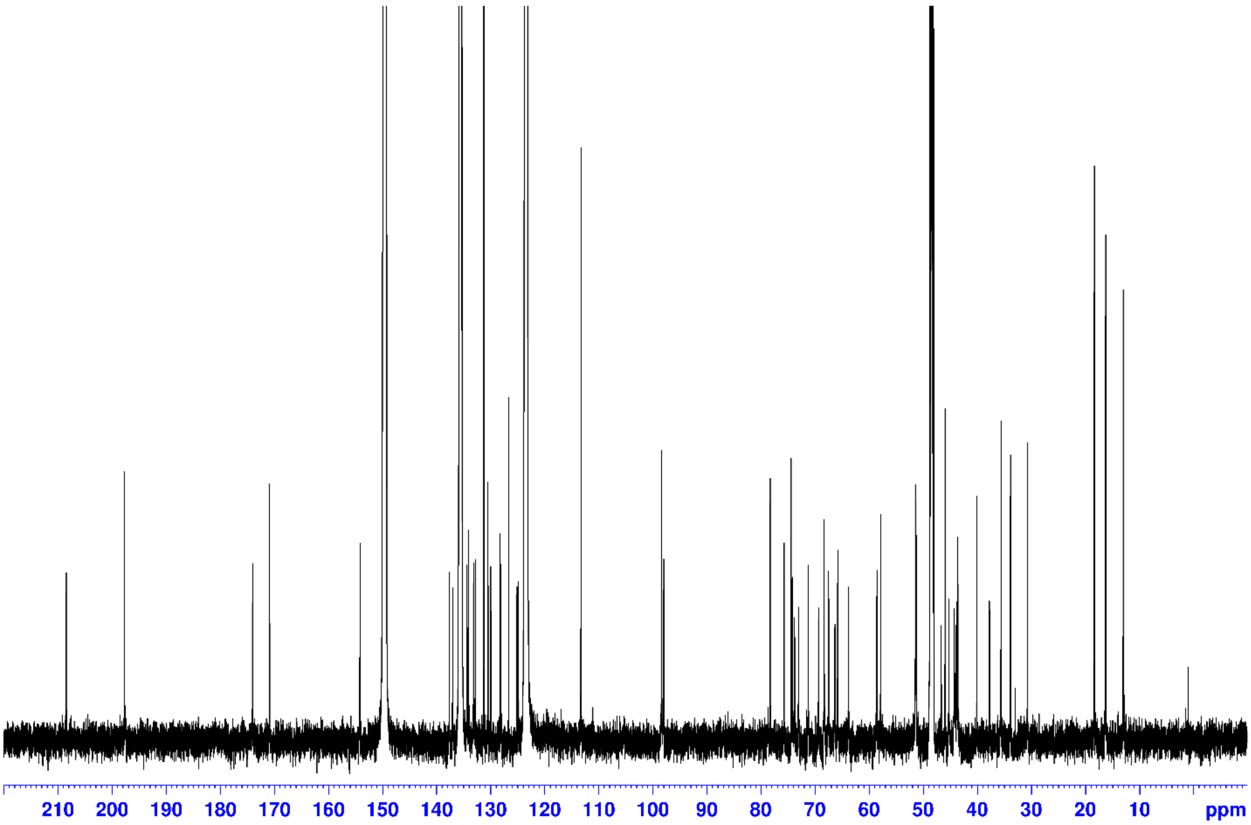

Figure S4. ^{13}C NMR spectrum of mepartricin B.

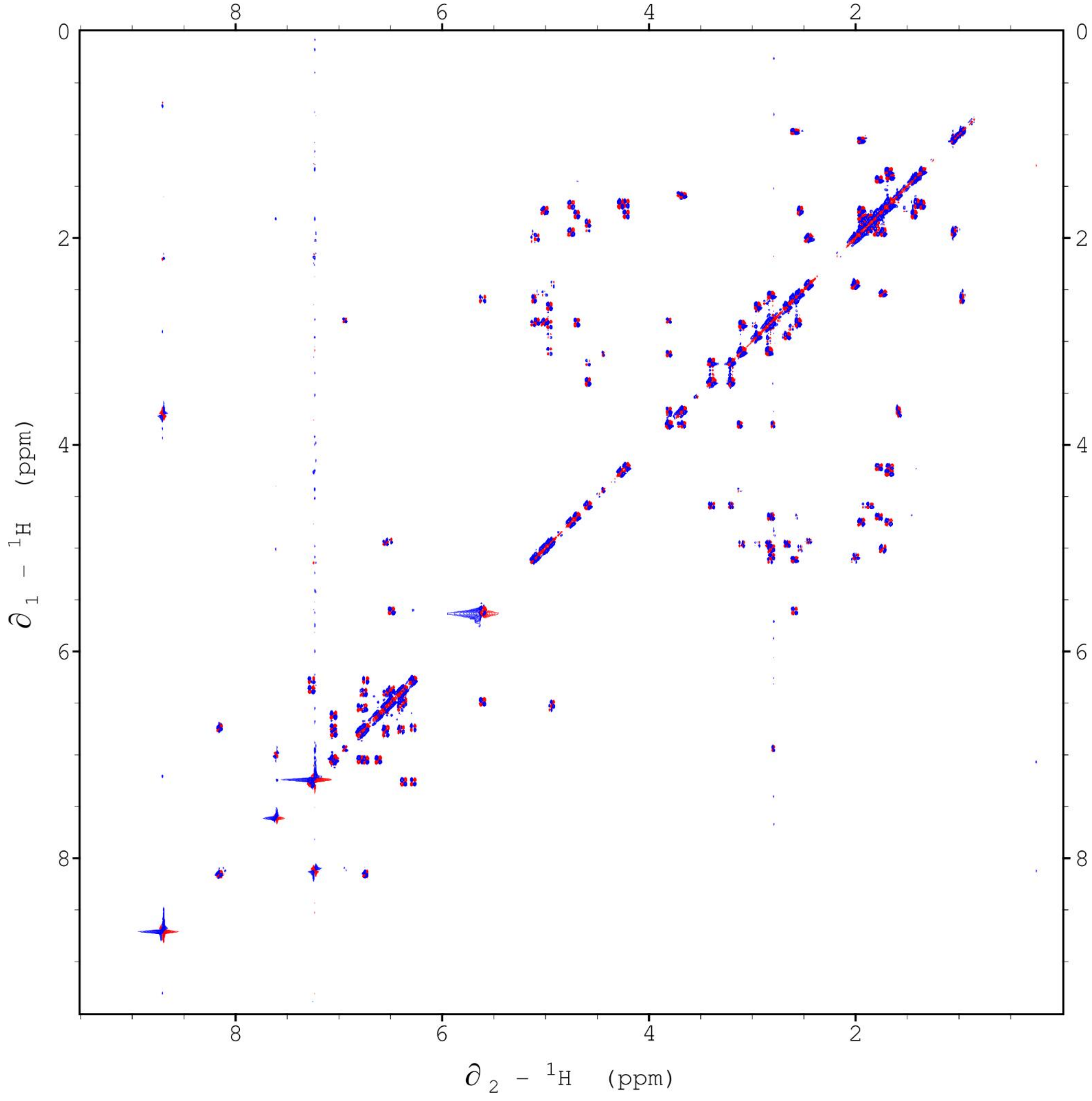

Figure S5. 2D-^{1}H,^{1}H-DQF-COSY spectrum of mepartricin A.

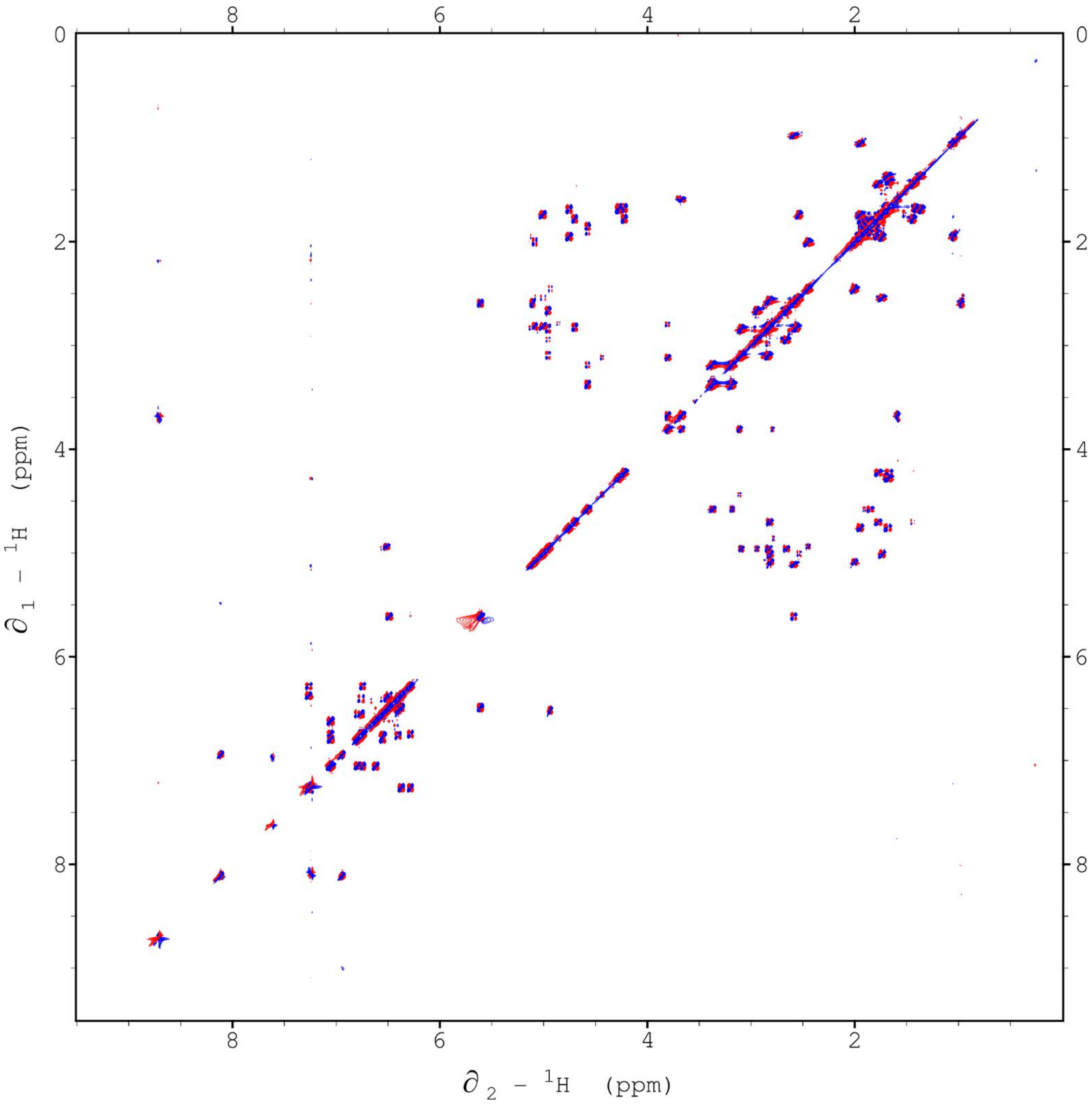

Figure S6. 2D-^{1}H,^{1}H-DQF-COSY spectrum of mepartricin B.

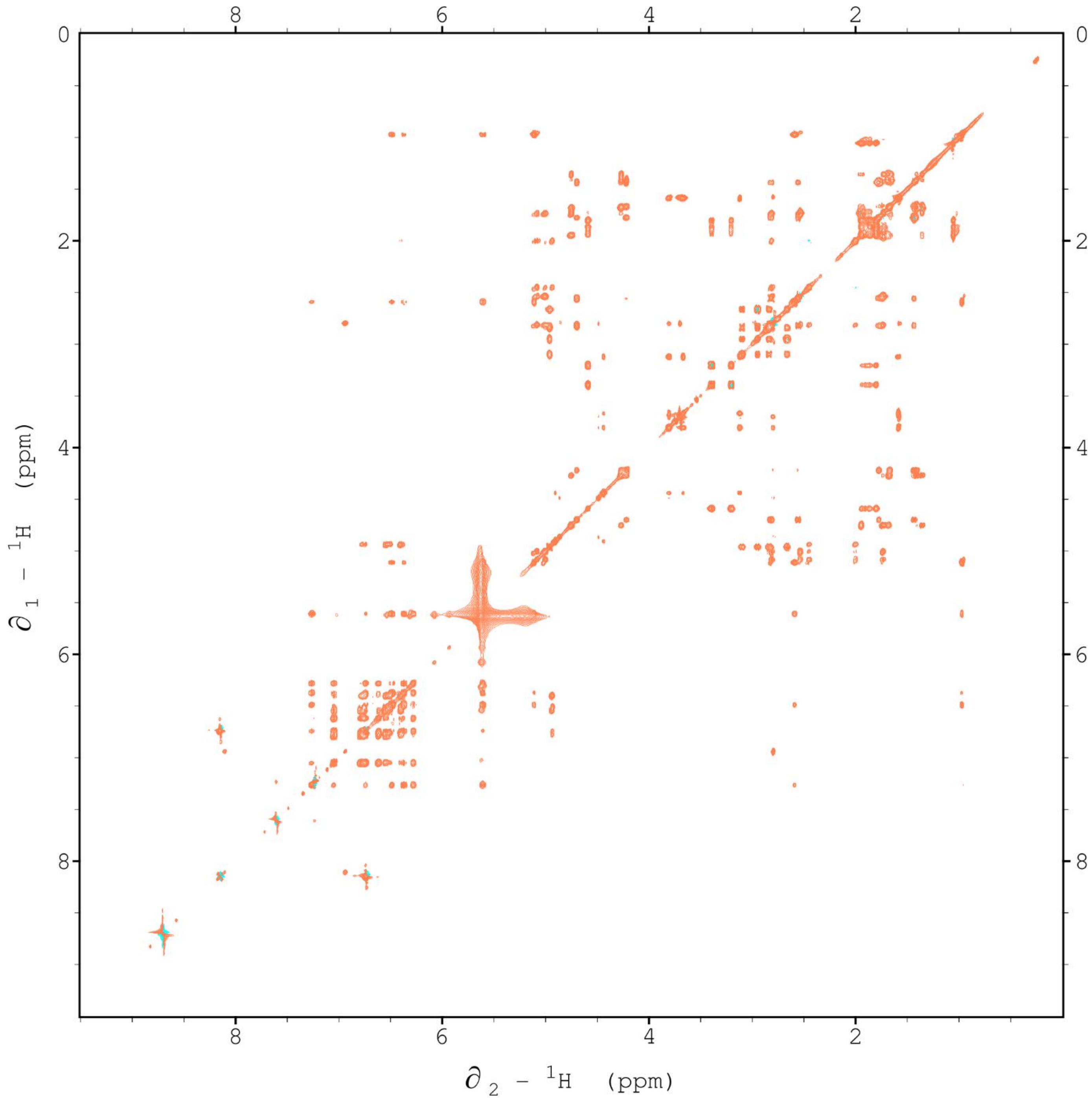

Figure S7. 2D-^{1}H,^{1}H-TOCSY spectrum of mepartricin A.

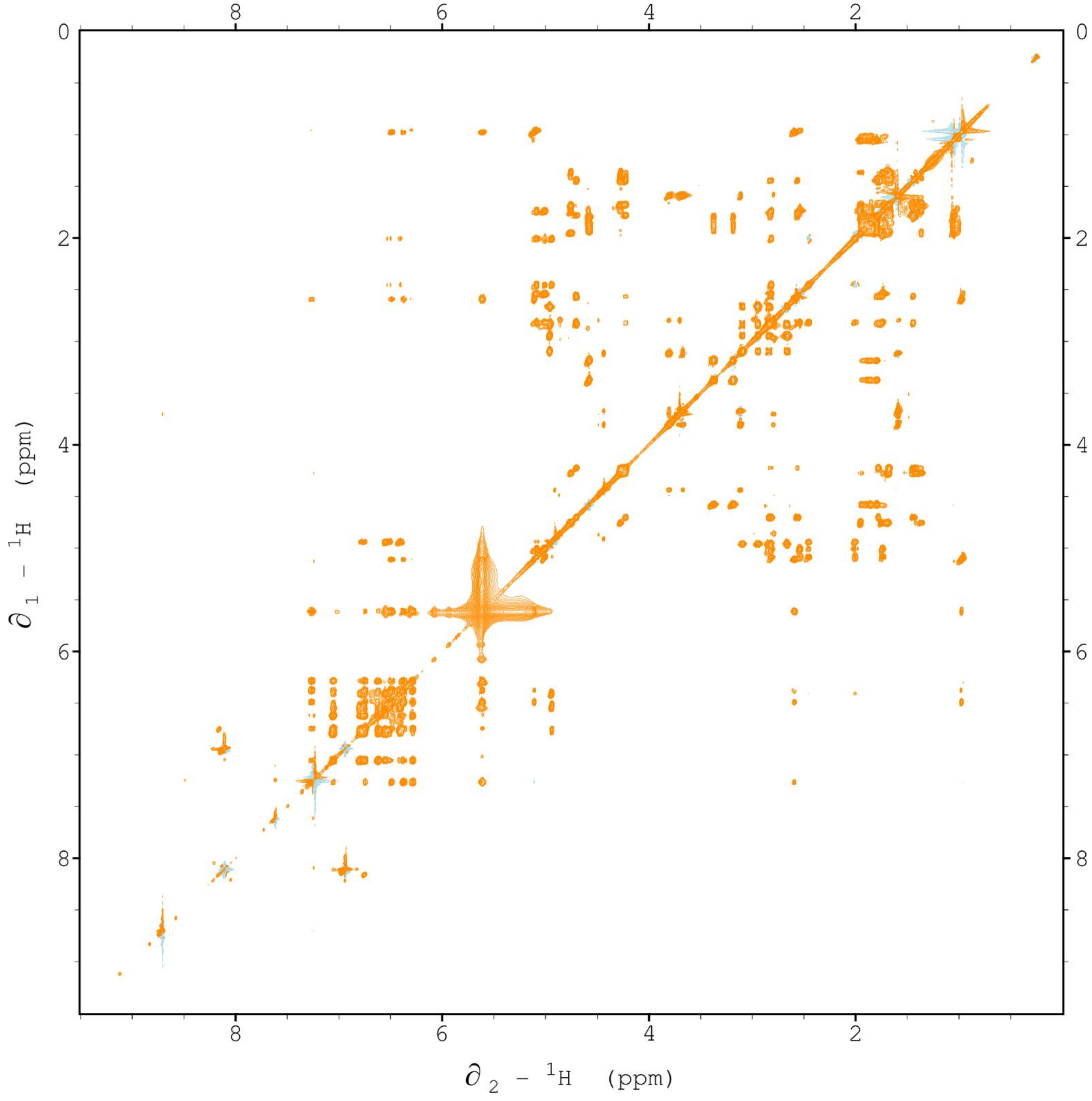

Figure S8. 2D-^{1}H,^{1}H-TOCSY spectrum of mepartricin B.

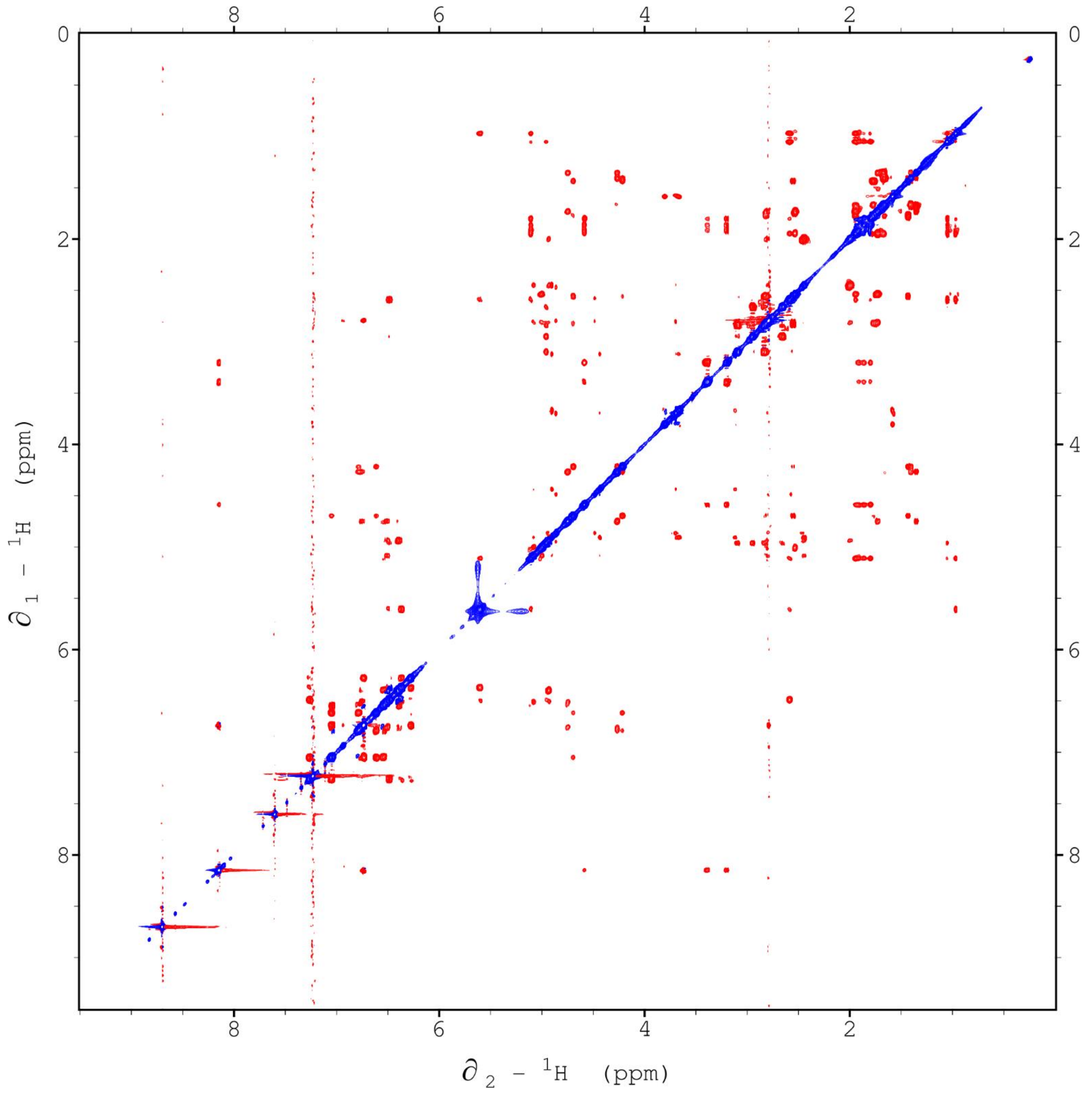

Figure S9. 2D-^{1}H,^{1}H-ROESY spectrum of mepartricin A.

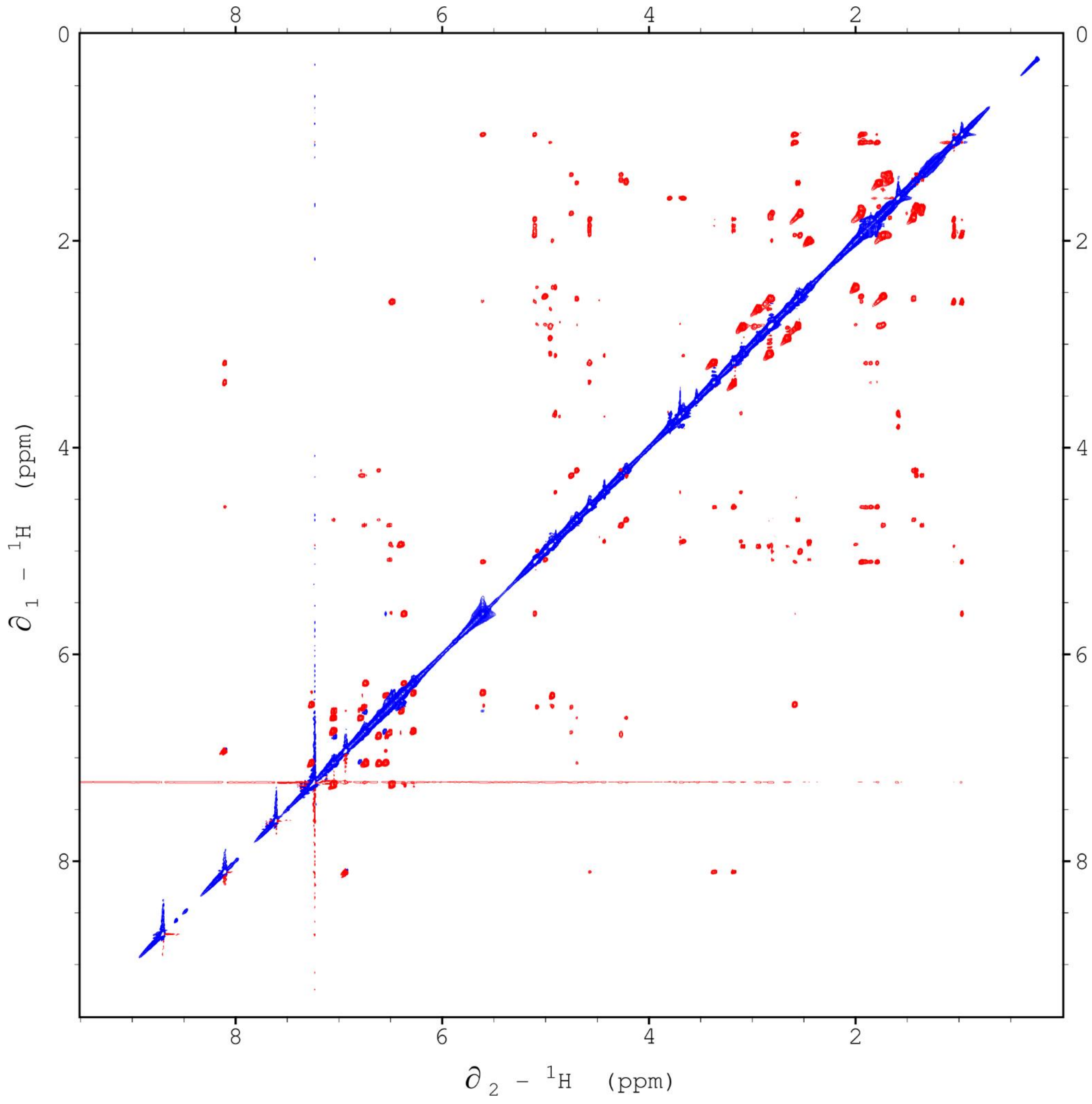

Figure S10. 2D-^{1}H,^{1}H-ROESY spectrum of mepartricin B.

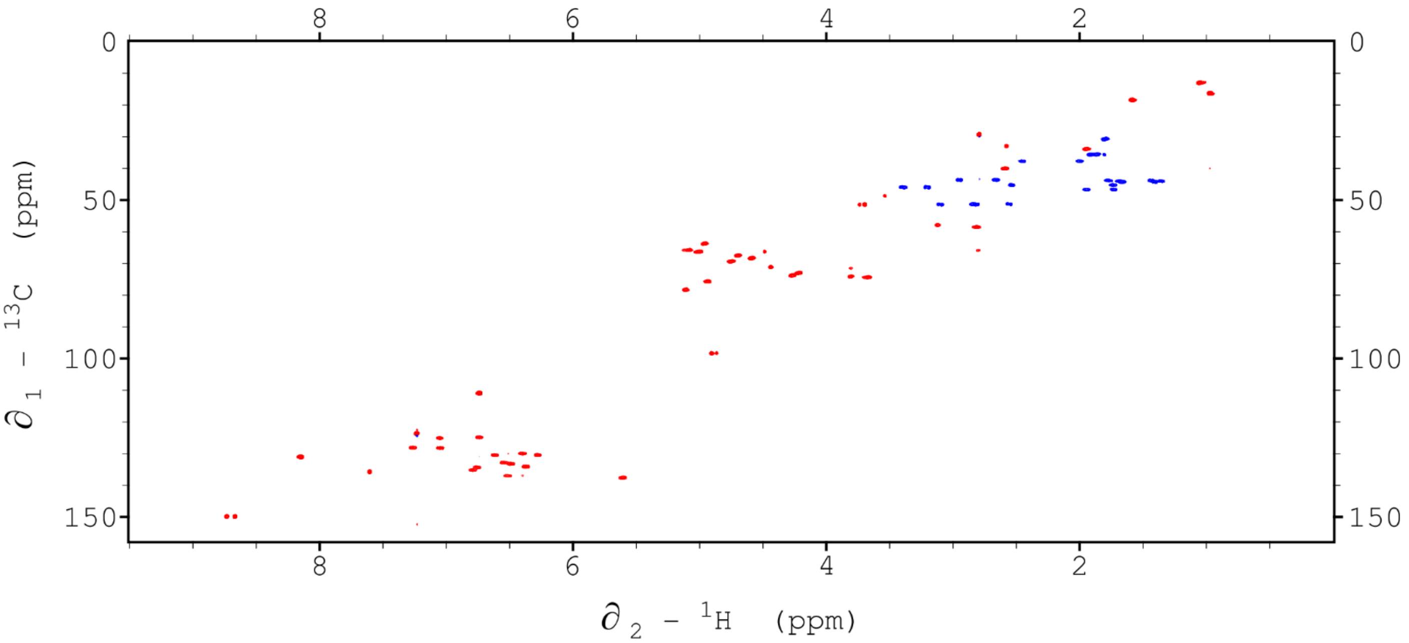

Figure S11. Edited 2D-^{1}H,^{13}C-HSQC spectrum of mepartricin A.

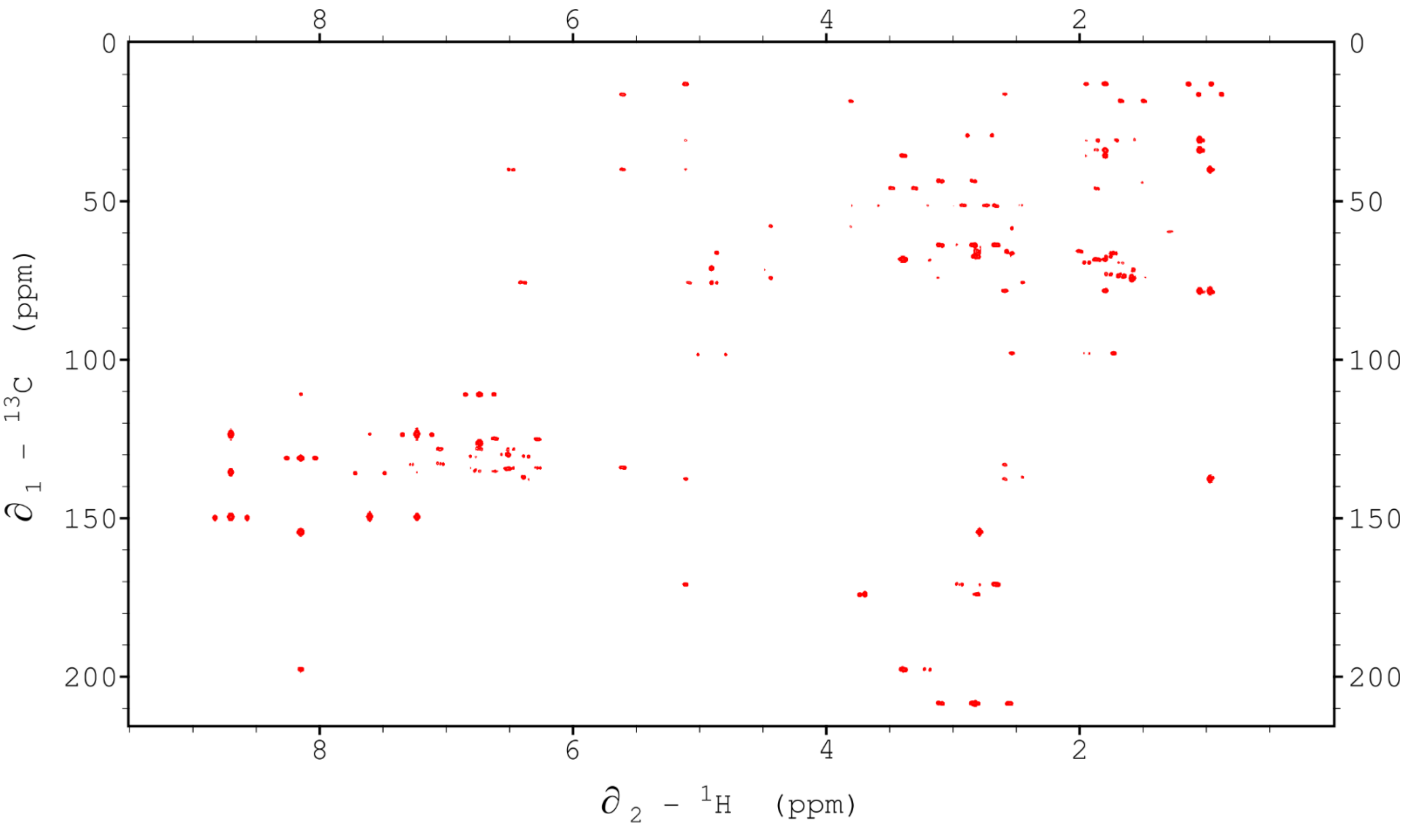

Figure S12. 2D-^{1}H,^{13}C-HMBC spectrum of mepartricin A.

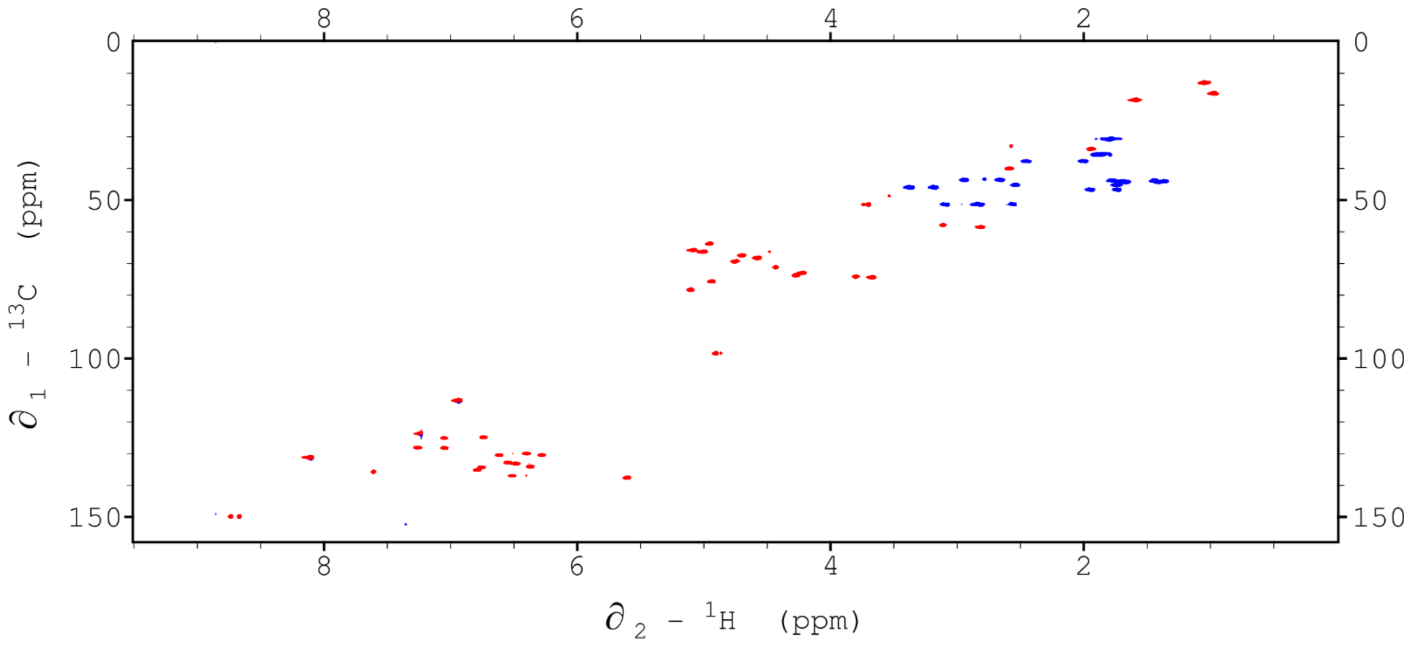

Figure S13. Edited 2D-^{1}H,^{13}C-HSQC spectrum of mepartricin B.

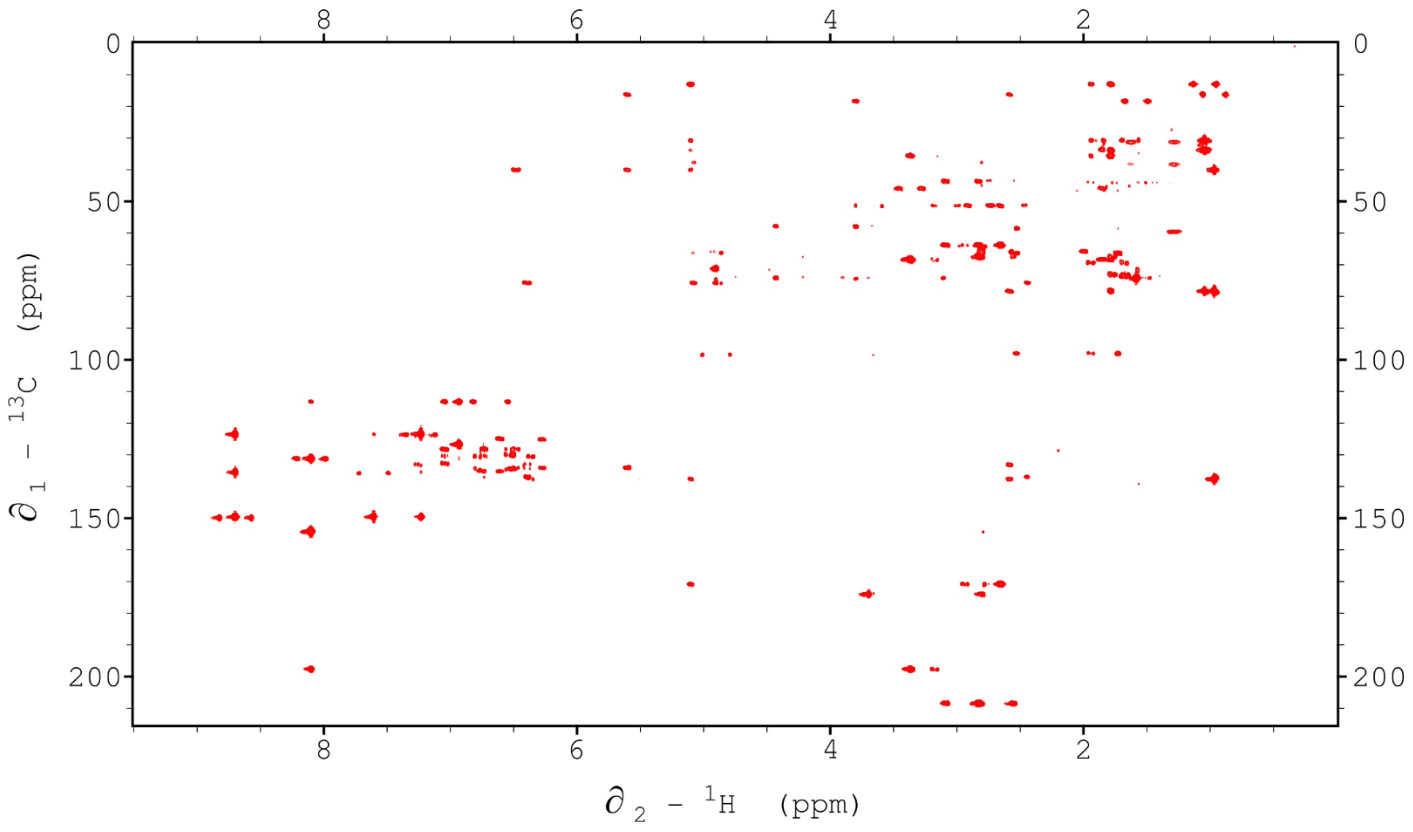

Figure S14. 2D-^{1}H,^{13}C-HMBC spectrum of mepartricin B.

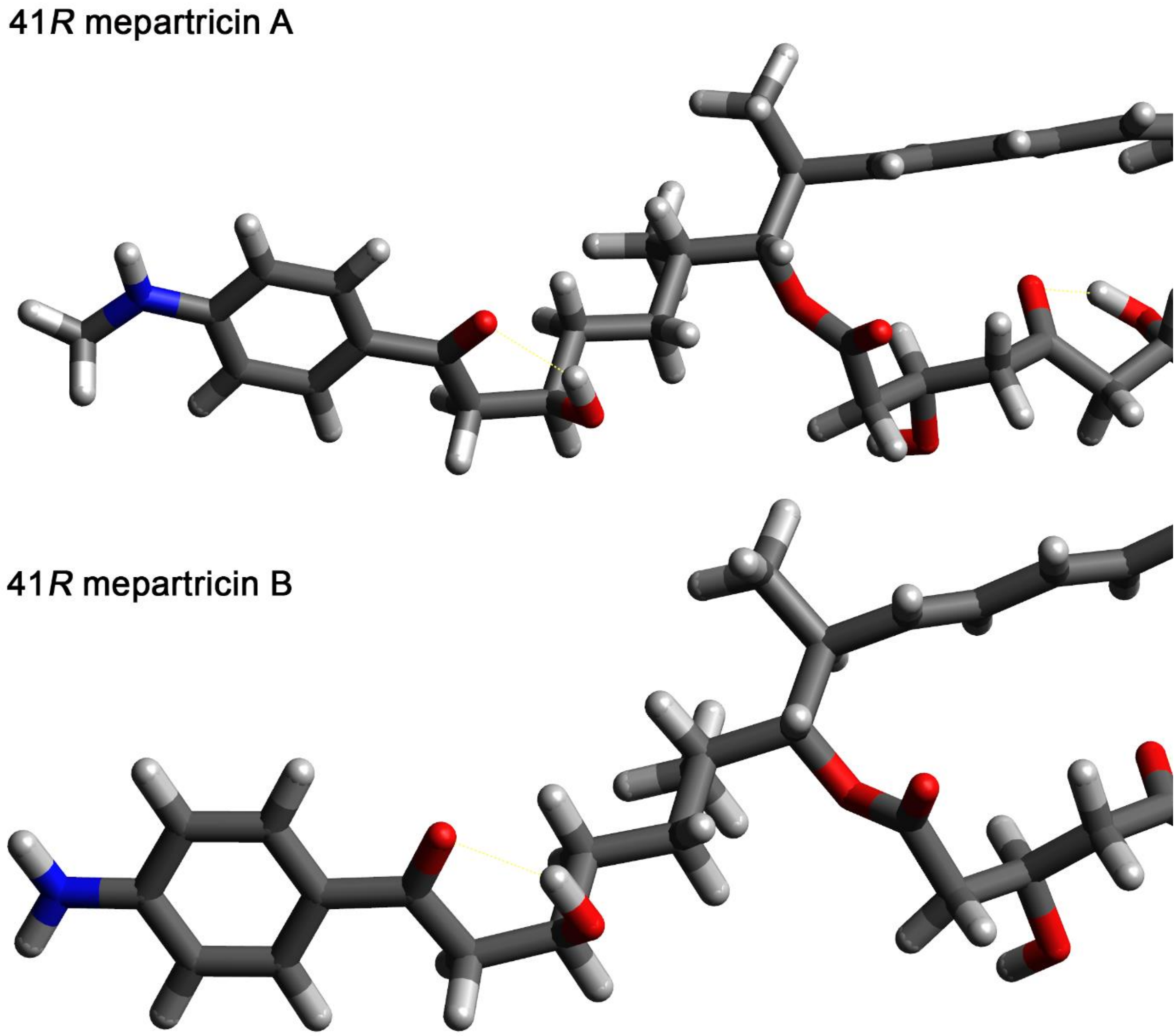

Figure S15. Average structures of the alkyl-aromatic sidechains of the possible 41*R*-epimers of mepartricins A and B.

41*S* mepartricin A

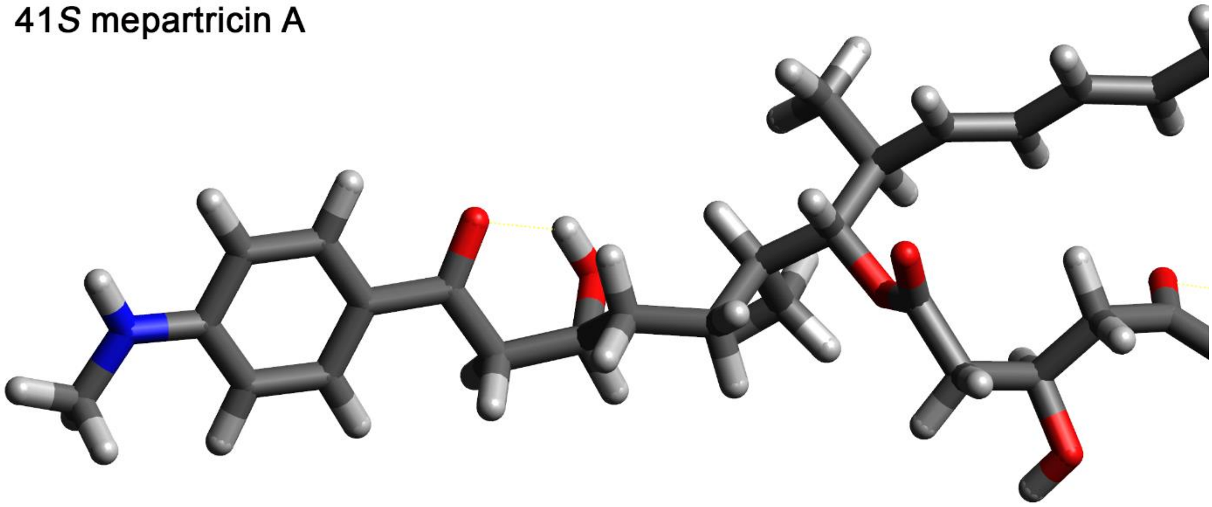

41*S* mepartricin B

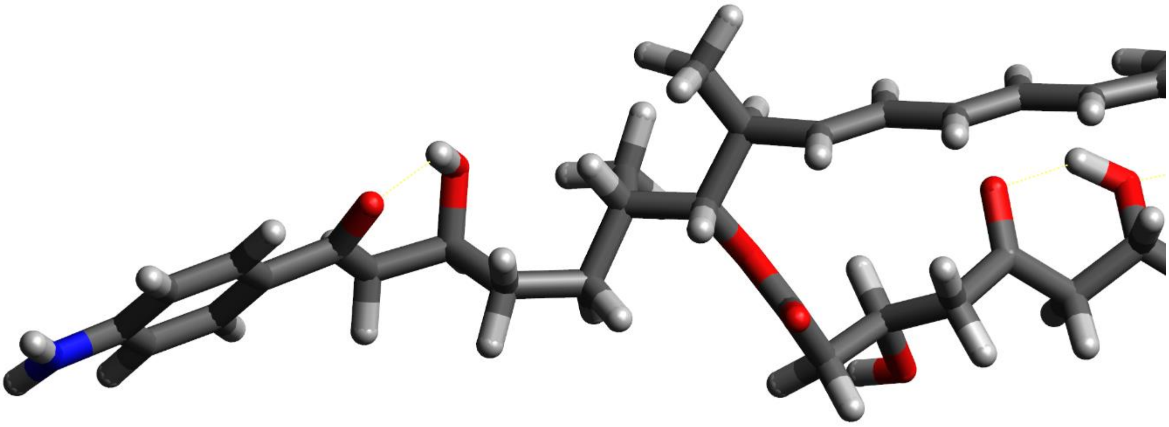

Figure S16. Average structures of the alkyl-aromatic sidechains of the possible 41*S*-epimers of mepartricins A and B.
